# Dapsone‐Induced Heinz Body Hemolytic Anemia: A Case Report

**DOI:** 10.1002/ccr3.70430

**Published:** 2025-04-15

**Authors:** Tyler Edwards, Monica Montes‐Rivera, Lucy Fu

**Affiliations:** ^1^ Northwestern Memorial Hospital Chicago Illinois USA; ^2^ Baylor College of Medicine Houston Texas USA

**Keywords:** dapsone, glucose‐6‐phosphate dehydrogenase (G6PD) deficiency, Heinz bodies, Heinz body hemolytic anemia, hemolytic anemia

## Abstract

Patients who develop acute anemia, without evidence of blood loss, should have a hemolysis workup including a medication review in case this could be contributing. In our patient's case, workup was significant for Heinz body hemolytic anemia due to home dapsone. Patient's anemia ultimately resolved with drug cessation.

## 1 Introduction

Acute anemia is a common issue among hospitalized patients with a broad differential diagnosis. Acute blood loss is often first suspected, especially in patients such as the one presented, in the setting of recent trauma, anticoagulation initiation, and advanced age. This often leads to further invasive testing, which is not helpful in patients with ongoing hemolysis and delays diagnosis. Dapsone‐induced Heinz body hemolytic anemia is a rare entity that requires a high index of suspicion to diagnose. As drug cessation is the only treatment, prompt recognition is essential to prevent worsening anemia. Also, testing for G6PD deficiency prior to starting dapsone can prevent significant potential morbidity. We hope to raise awareness among health care professionals to improve diagnostic suspicion and increase recognition.

## 2 Case History/Examination

An 85‐year‐old Caucasian male presented with acute shortness of breath of one‐day duration. His past medical history included basal cell carcinoma, essential hypertension, and myasthenia gravis. His medications were amlodipine, losartan, pantoprazole, prednisone, and dapsone. He sustained a mechanical fall about 1 month prior to presentation but did not seek any medical care.

Since then, he endorses being significantly less mobile around his home due to low back pain from the fall. His review of systems was otherwise negative. He denied fever/chills, cough, sputum production, sick contacts, recent travel, orthopnea, leg swelling, or overt signs of bleeding.

On presentation, vitals were notable for: temperature of 37°C, blood pressure of 126/76 mmhg, pulse was 78 beats per minute, oxygen saturation via pulse oximetry (SpO2) of 95% on room air, and respiratory rate of 18 breaths per minute. His physical examination, including pulmonary and cardiac exam, was unremarkable. Initial laboratory results showed a hemoglobin level of 8.7 g/dL (reference range 11.7–15.0 g/dL) from baseline of 13 g/dL with MCV of 108.

Platelets and white blood cell count were within normal limits. BMP was without abnormality. An EKG showed normal sinus rhythm and was without ischemic changes. Chest x‐ray did not reveal any acute abnormalities.

Computed tomography angiography of the chest showed new pulmonary emboli within the bifurcation of the right upper lobe pulmonary artery extending into the origin of the segmental branches and the right middle lobe segmental artery. The patient was started on anticoagulation with a heparin drip.

After initiation of anticoagulation, the patient's hemoglobin continued to decline over the next 2 days with a nadir of 7 g/dL. There were no signs of overt bleeding.

Given the recent fall, hemoglobin drop, and initiation of anticoagulation, there was concern for intra‐abdominal or retroperitoneal blood loss. Computed tomography of the abdomen and pelvis was negative for any source of bleeding. The patient's vital signs remained stable during this time.

Further laboratory workup revealed an indirect bilirubin of 2.5 mg/dL (reference range 0.2–0.8 mg/dL) which was noted to be present 2 months prior to presentation. Given this persistent indirect hyperbilirubinemia, a hemolysis workup was initiated.

**FIGURE 1:**
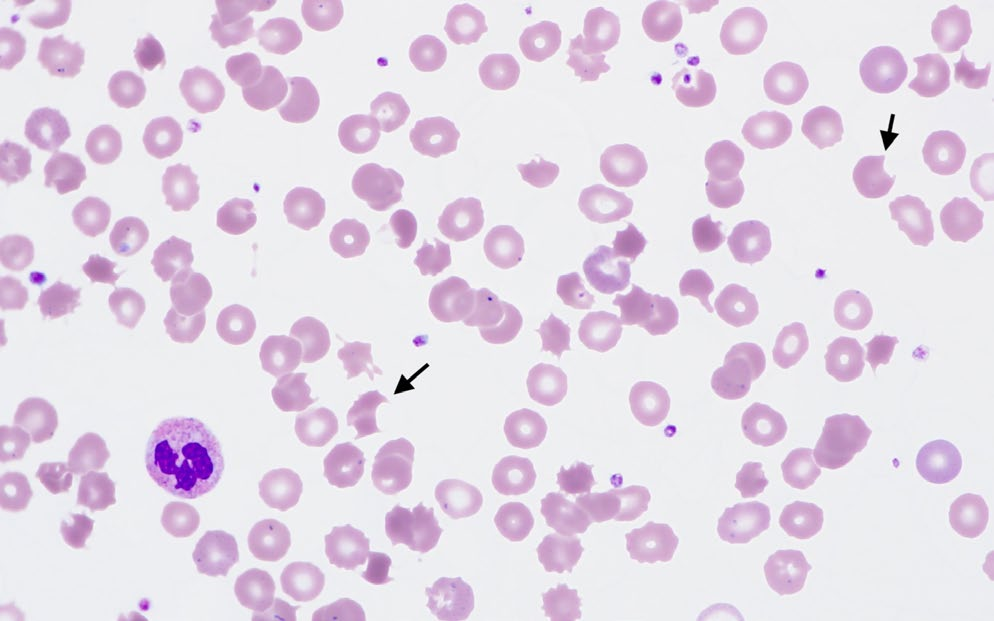
Patient's peripheral smear demonstrating bite cells as indicated by the black arrows.

Results included lactate dehydrogenase DH 460 U/L (reference rage 90–180 U/L), haptoglobin < 30 mg/dL (reference range 30–200 mg/dL), INR within normal limits, and an absolute reticulocyte count of 247 K/UL (reference range 27–110 K/UL). Direct antiglobulin test was negative. Hematology was consulted for concern for hemolysis. A peripheral smear showed bite cells (as shown in Figure 1).

## 3 Differential Diagnosis

Autoimmune Hemolytic Anemia, acute blood loss anemia, Hereditary Spherocytosis, Disseminated Intravascular Coagulation, Paroxysmal Nocturnal Hemoglobinuria.

## 4 Conclusion/Results

Upon further review of the patient's history, it was noted that 2 months prior to presentation, the patient had a myasthenia gravis flare and was started on a high dose of prednisone, along with dapsone for Pneumocystis Jiroveci pneumonia prophylaxis.

**FIGURE 2:**
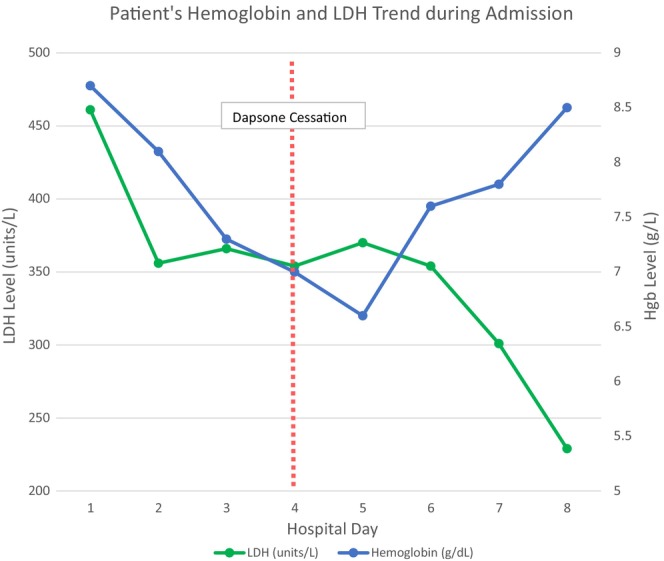
Patient's hemoglobin and LDH trend during hospital admission. Timing of dapsone cessation is denoted by vertical red line.

Based on the review of the peripheral smear and the patient's history, he was diagnosed with Heinz body hemolytic anemia triggered by dapsone. G6PD deficiency was questioned given the severity of hemolysis. G6PD level came back normal but this could be falsely normal in setting of acute hemolysis. Also, patient had not had G6PD level checked prior to starting dapsone. Dapsone was discontinued and patient's hemoglobin improved to 8.5 g/dL over the next 2 days. Also, there was a simultaneous decrease in LDH and indirect bilirubin at the time of discharge (as shown in Figure 2). Hemoglobin was repeated about 6 months later with further improvement to 12.5 g/dL, which was around the patient's baseline. Upon discharge from the hospital, the patient was instructed to establish care with a primary care doctor, so his G6PD level could be retested 3 months later; however, he was lost to follow‐up and did not have this test repeated.

We have presented a case of a patient who developed severe anemia upon initiation of systemic anticoagulation but was found to actually have hemolytic anemia due to dapsone. This case highlights the importance of maintaining a broad differential when working up anemia and avoiding anchoring solely on blood loss. When starting a patient on dapsone, G6PD level should be checked prior to initiation. Patients started on dapsone can develop severe hemolytic anemia if they have undiagnosed G6PD deficiency. Lastly, when initiating dapsone, patients should have a hemoglobin check once weekly for the first month to monitor for developing hemolytic anemia. This patient did not have routine hemoglobin monitoring performed, which likely delayed his diagnosis.

Drug cessation is the only treatment for dapsone induced hemolysis. Therefore, prompt recognition is crucial to prevent excess morbidity, and this case serves to raise awareness among healthcare professionals of this disease entity to increase recognition and reduce delay in diagnosis.

## 5 Discussion

Heinz body hemolytic anemia is a rare condition characterized by the presence of Heinz bodies in red blood cells and associated hemolysis. Heinz bodies are denatured hemoglobin precipitates that form within red blood cells as a result of oxidative damage to the hemoglobin molecule and then bind to the erythrocyte cell membrane.

Factors contributing to Heinz body formation include exposure to oxidant drugs (such as dapsone), certain infections, and metabolic disorders [1]. In this patient's case, dapsone was the triggering exposure. Life‐threatening cases of hemolytic anemia can be seen in patients with underlying G6PD deficiency.

Dapsone induces hemolysis through multiple mechanisms. These include oxidative stress and membrane protein alterations [2]. Dapsone is metabolized to dapsone hydroxylamine (DDS‐NOH), which is responsible for the hemolytic activity that can be observed. DDS‐NOH generates reactive oxygen species within red blood cells, which induce oxidative stress.

This leads to the formation of disulfide‐linked hemoglobin adducts on membrane skeletal proteins (Heinz bodies) and compromises the structural integrity of red blood cells [3]. This alone can lead to intravascular hemolysis.

This increased oxidative stress also triggers a process known as eryptosis, or programmed cell death in erythrocytes [4]. These eryptotic cells are recognized and phagocytosed by circulating macrophages, which act to decrease the severity of hemolysis. Also, DDS‐NOH causes alterations in membrane proteins, such as band 3 protein aggregation and increased tyrosine phosphorylation [5].

These changes lead to the binding of autologous antibodies to the red blood cell membrane. Both oxidative stress and membrane protein alteration lead to increased splenic destruction and extravascular hemolysis of affected red blood cells [6].

The spleen's removal of formed Heinz bodies from the erythrocyte cell membrane leads to the creation of the characteristic bite cell, as seen on our patient's peripheral blood smear.

The clinical presentation of Heinz body hemolytic anemia can vary, ranging from mild to severe anemia, jaundice, and symptoms related to anemia. It typically takes about 2 weeks for hemolytic anemia to develop after starting dapsone.

Laboratory findings often include the presence of Heinz bodies on peripheral blood smear, an increased reticulocyte count, and evidence of hemolysis, such as elevated LDH and decreased haptoglobin levels.

G6PD level is often falsely normal during times of acute hemolysis due to the increased presence of reticulocytes, which have higher G6PD activity compared to older red blood cells. The G6PD level needs to be rechecked 3 months after the hemolytic event. Worsening anemia after initiation of anticoagulation is a relatively common complication.

In cases such as this, patients often undergo extensive GI evaluation with imaging and endoscopy to identify a source of blood loss. In this patient's case, his worsening anemia and anticoagulation initiation were only coincidental. The only initial clue to the underlying hemolytic process was a mildly elevated indirect bilirubin, which led to further investigation to identify the cause.

Specifically, in this case, prompt recognition of hemolytic anemia due to dapsone was essential. For this diagnosis, drug cessation is crucial to prevent further hemolysis and the continued worsening of hemoglobin levels.

Further anchoring on blood loss from the GI tract would have led to unnecessary and invasive procedures, all while the patient's clinical status would have worsened as the underlying pathology would not have been addressed. This case demonstrates the importance of maintaining a broad differential diagnosis during the workup of anemia and further working up lab abnormalities that do not fit the initial diagnosis.

## Author Contributions

**Tyler Edwards:** conceptualization, writing – original draft, writing – review and editing. **Monica Montes‐Rivera:** writing – original draft. **Lucy Fu:** investigation, resources, visualization.

## Consent

Written informed consent was obtained from the patient to publish this report in accordance with the journal's patient consent policy.

## Conflicts of Interest

The authors declare no conflicts of interest.

## Data Availability

All data necessary for this case report are available as part of this article, and no additional source data are required.
